# Activation of c-Met in cancer cells mediates growth-promoting signals against oxidative stress through Nrf2-HO-1

**DOI:** 10.1038/s41389-018-0116-9

**Published:** 2019-01-15

**Authors:** Samik Chakraborty, Murugabaskar Balan, Evelyn Flynn, David Zurakowski, Toni K. Choueiri, Soumitro Pal

**Affiliations:** 1 Division of Nephrology, Boston Children’s Hospital, Boston, MA 02115 USA; 2 Harvard Medical School, Boston, MA 02115 USA; 3 Department of Anesthesiology, Boston Children’s Hospital, Boston, MA 02115 USA; 4 Dana Farber Cancer Institute, Boston, MA 02115 USA

**Keywords:** Apoptosis, Cancer therapeutic resistance

## Abstract

Any imbalance between reactive oxygen species (ROS) generation and the anti-oxidant capacity lead to cellular oxidative stress. Many chemotherapeutic agents mediate their cytotoxic functions through the generation of ROS. c-Met, a receptor tyrosine kinase, is over-expressed in renal cancer and plays very crucial role(s) in its growth and survival. Here, we show that c-Met activation protected renal cancer cells from ROS, oxidative stress and cytotoxicity induced by the anti-cancer agent sorafenib (used for renal cancer treatment); and it markedly attenuated sorafenib-induced DNA damage. Activated c-Met promoted the anti-apoptotic proteins (Bcl-2 and Bcl-xL) and inhibited apoptotic cleaved caspase-3. We found that the cytoprotective function of c-Met against sorafenib-induced ROS generation and apoptosis was mediated primarily through the activation of anti-oxidant Nrf2-HO-1. c-Met promoted the nuclear localization of Nrf2 and hindered its binding with the inhibitory protein Keap1. Silencing of Nrf2 attenuated the protective action of c-Met against sorafenib-induced oxidative stress. To evaluate the physiological significance of our findings, in a tumor xenograft model, we observed that a combination treatment with pharmacological inhibitors of c-Met and it’s anti-oxidant downstream effecter HO-1 markedly reduced the growth of renal tumor in vivo; it increased the oxidative stress, DNA damage and apoptotic markers in the tumor xenografts, along with reduced tumor vessel density. Our observations indicate that the c-Met-Nrf2-HO-1 pathway plays a vital role in relieving ROS-mediated oxidative stress of renal tumors. Targeting this pathway can significantly increase the oxidative stress to promote apoptotic death of cancer cells.

## Introduction

Kidney or renal cancer is one of the ten most common type of cancers in both men and women; also, it has very limited treatment options in advanced stages^1–3^. Also, most of the renal cancer patients develop resistance against chemotherapeutic drug treatments. c-Met, a receptor tyrosine kinase, is over-expressed in clear cell as well as in papillary renal cell carcinoma (RCC)^2,4^, and recent studies suggest that c-Met can be a potential therapeutic target. After being phosphorylated by its ligand the hepatocyte growth factor (HGF), c-Met can induce a broad spectrum of biological pathways involved in tumor growth^2,4,5^. However, how the c-Met activation can modulate the oxidative stress and cytotoxicity induced by chemotherapeutic agents, like sorafenib, has not been thoroughly explored.

Reactive oxygen species (ROS) are usually hyperactive small molecules that last for short period of time^6,7^. ROS at low to modest concentrations are considered to be crucial for the regulation of normal physiological and developmental functions^8^. Oxidative stress is an outcome resulting from a disruption of balance between ROS generation and the cellular anti-oxidant capacity^6^. Accumulation of relatively higher than normal physiological levels of ROS promotes the oxidative damage of DNA^9^. The mitochondrial electron transport chain^10^ and the NADPH oxidases (NOX) are major players of intracellular ROS generation^11^. ROS have a double edged role in cancer cells; at lower concentrations it facilitates the growth and proliferation, whereas in higher concentrations it orchestrates the killing of the tumor cells^10^. Several chemotherapeutic agents exert their cytotoxic effects on cancer cells through increased ROS generation^12^. However, cancer cells can bypass ROS-mediated killing by utilizing the cellular anti-oxidant system^12^.

Heme oxygenase-1 (HO-1), an anti-oxidant enzyme, promotes the breakdown of heme into carbon monoxide (CO), biliverdin and ferrous iron^5,13,14^. Previously, we showed that the activation of c-Met induces HO-1 overexpression that favors renal cancer cell survival^5,15^. The transcription of HO-1 is an intricate process and regulated through the positive modulator nuclear factor E2-related factor 2 (Nrf2) and the negative modulator Bach-1^5,16^. The expression of HO-1 is induced when the heterodimers of Nrf2 and small Maf proteins get bound to Maf recognition elements^17^. On other hand, the heterodimers of Bach-1 and small Maf proteins downregulate HO-1 expression^18^. The transcription factor Nrf2 is sensitive according to the cellular redox state^18^. When there is no oxidative stress, its reactivity is suppressed through Kelch-like erythroid-derived cap-n-collar (CNC) homology (ECH)-associated protein 1 (Keap1)^19^. After being released from Keap1, Nrf2 is migrated to nucleus and interacts with Maf proteins^19^. The formed complex then gets bound to the anti-oxidant-response element (ARE) and induces the overexpression of anti-oxidant/cytoprotective molecules, like HO-1, superoxide dismutase and NQO1^20^.

As discussed earlier, the mechanism of action of several chemotherapeutic agents is through increased ROS production and elevated oxidative stress in cancer cells^12^. Sorafenib is a multikinase inhibitor, which can downregulate tumor proliferation and angiogenesis^21,22^. Sorafenib is currently being used in the treatment for metastatic RCC and other cancers^21–23^. Sorafenib is known to generate ROS and inhibit mitochondrial respiration along with the interruption of cellular glycolysis^23^. It has recently been shown that sorafenib can disrupt the mitochondrial membrane potential in cancer cells, leading to increased ROS, and thereby breaking the resistance to TRAIL-induced apoptosis^24^. However, the susceptibility of sorafenib-induced ROS generation against c-Met-Nrf2-HO-1 cytoprotective axis is yet to be explored.

In the present work, it has been demonstrated that the activation of c-Met pathway in renal cancer downregulates sorafenib-induced ROS generation through the modulation of Nrf2-HO-1, and it inhibits cancer cell death. Our findings also suggest a mechanism for c-Met-induced chemoresistance of cancer cells.

## Results

### Induction of c-Met mediates protection of renal cancer cells from chemotherapeutic drug-induced ROS generation and apoptosis

**Fig. 1:**
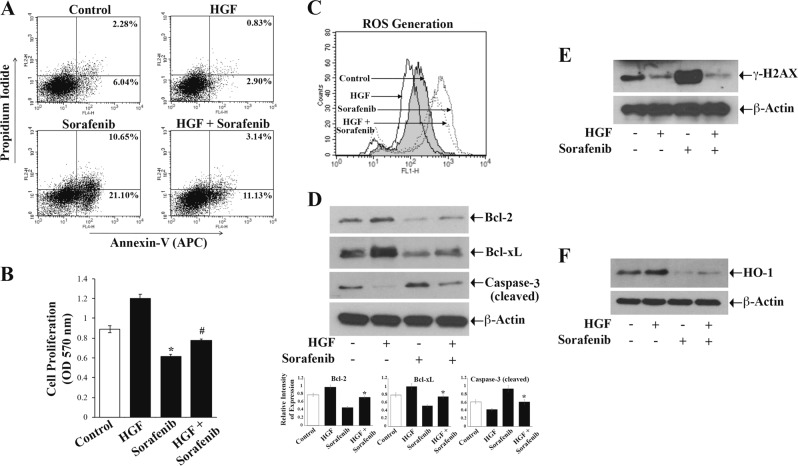
**c-Met induction protects the renal cancer cells from sorafenib-induced ROS generation, DNA damage, and apoptosis.** **a–f** 786-O cells were subjected to combination treatments with HGF (50 ng/ml), sorafenib (10 μM) or only vehicle for 24 h. **a** Apoptotic cell deaths were measured by annexin V (APC) and propidium iodide staining through flow cytometry. **b** Cell proliferation was measured by MTT assay. **c** Following treatment, cells were stained by using oxidative stress detection reagent and checked for endogenous reactive oxygen species (ROS) through flow cytometry. **d**–**f** Fractions of cellular lysates were utilized to analyze levels of Bcl-2, Bcl-xL, cleaved Caspase-3 (**d**); γ-H2AX (**e**) and HO-1 (**f**) by Western blot. β-Actin was used as an internal control (**d**–**f**). In **a** and **c**–**f**: Results are representative of three different experiments. In **b**: The columns are the mean ± S.D. of triplicate readings from two different samples. *p *<* 0.05 compared with vehicle-treated control cells, and #*p* < 0.05 compared with cells treated with sorafenib alone. In **d**, the *bar graphs* under the Western blots represent the relative expression Bcl-2/Bcl-xL/Caspase-3 (cleaved) by densitometry, where the signals were normalized to the expression of an internal control β-Actin. The *columns* represent the average ± S.D of relative intensities from three different blots. *p *<* 0.05 compared with cells treated with sorafenib alone

We recently reported that c-Met activation can promote the cytoprotection and survival of renal cancer cells^5^. Here, we wanted to check if c-Met-mediated signals can protect renal cancer cells (786-O and ACHN) from cytotoxic effects of the chemotherapeutic agent sorafenib. We found that sorafenib treatment increased the apoptosis of both 786-O and ACHN cells compared with the control. However, after the activation of c-Met (following HGF treatment), there was a marked reduction of sorafenib-induced cellular apoptosis. In the presence of HGF, the total apoptotic (early + late) 786-O cells after sorafenib treatment decreased from (21.10% + 10.65%) = 31.75% to (11.13% + 3.14%) = 14.27% (Fig. 1a). Similarly, in the presence of HGF, the total apoptotic ACHN cells following sorafenib treatment decreased from 31.35% to 15.99% (Supplementary Fig. 1A). Interestingly, we observed that the inhibition of c-Met through XL-184 (a pharmacological inhibitor of c-Met) markedly increased renal cancer cell apoptosis after sorafenib treatment (Supplementary Fig. 2). We also found that sorafenib treatment downregulated cancer cell proliferation; and this was significantly prevented in the presence of HGF (Fig. 1b).

Sorafenib is known to exert its cytotoxic effect through ROS generation^23,25^. As the activation of c-Met promotes a protection against sorafenib-induced cytotoxicity, we next checked whether c-Met can modulate sorafenib-induced ROS generation in renal cell carcinoma (RCC) cells. As shown in Fig. 1c, in 786-O cells, sorafenib increased the cellular ROS. However, the treatment with HGF decreased sorafenib-induced ROS generation. Similar results were obtained in ACHN cells (Supplementary Fig. 1B). We also found that the treatment with HGF restored sorafenib-induced reduction of the anti-apoptotic factors Bcl-2 and Bcl-xL (Fig. 1d, *upper two panels*). Moreover, c-Met activation markedly inhibited sorafenib-induced increase of the pro-apoptotic cleaved caspase-3 (Fig. 1d, *third panel*).

One of the main events in chemotherapeutic agent-induced ROS generation and apoptotic death of cancer cells is the induction of a DNA damage response, which is characterized by the presence of DNA damage foci or γ–Histone2AX (γ–H2AX)^26–28^. Here, the expression of γ–H2AX was assayed in RCC cells following treatments with sorafenib and HGF. We observed that sorafenib markedly increased γ–H2AX; however, sorafenib-induced γ–H2AX expression was significantly downregulated in the presence of HGF (Fig. 1e). We have previously shown that the anti-oxidant HO-1 is an important effector for c-Met-mediated pathways in renal cancer growth. Here, we found that HGF treatment increased HO-1, while sorafenib decreased its expression compared with control; however, HO-1 was upregulated in sorafenib-treated cells in the presence of HGF compared with cells treated with sorafenib alone (Fig. 1f). Altogether, our observations indicated that the c-Met-HGF-HO-1 pathway plays critical role(s) in counteracting sorafenib-induced ROS generation and apoptosis; it can also be important in regulating chemotherapeutic drug-induced DNA damage repair in RCC cells.

### c-Met signaling modulates the redox sensitive Nrf2 to attenuate sorafenib-induced apoptotic index of the RCC cells

**Fig. 2:**
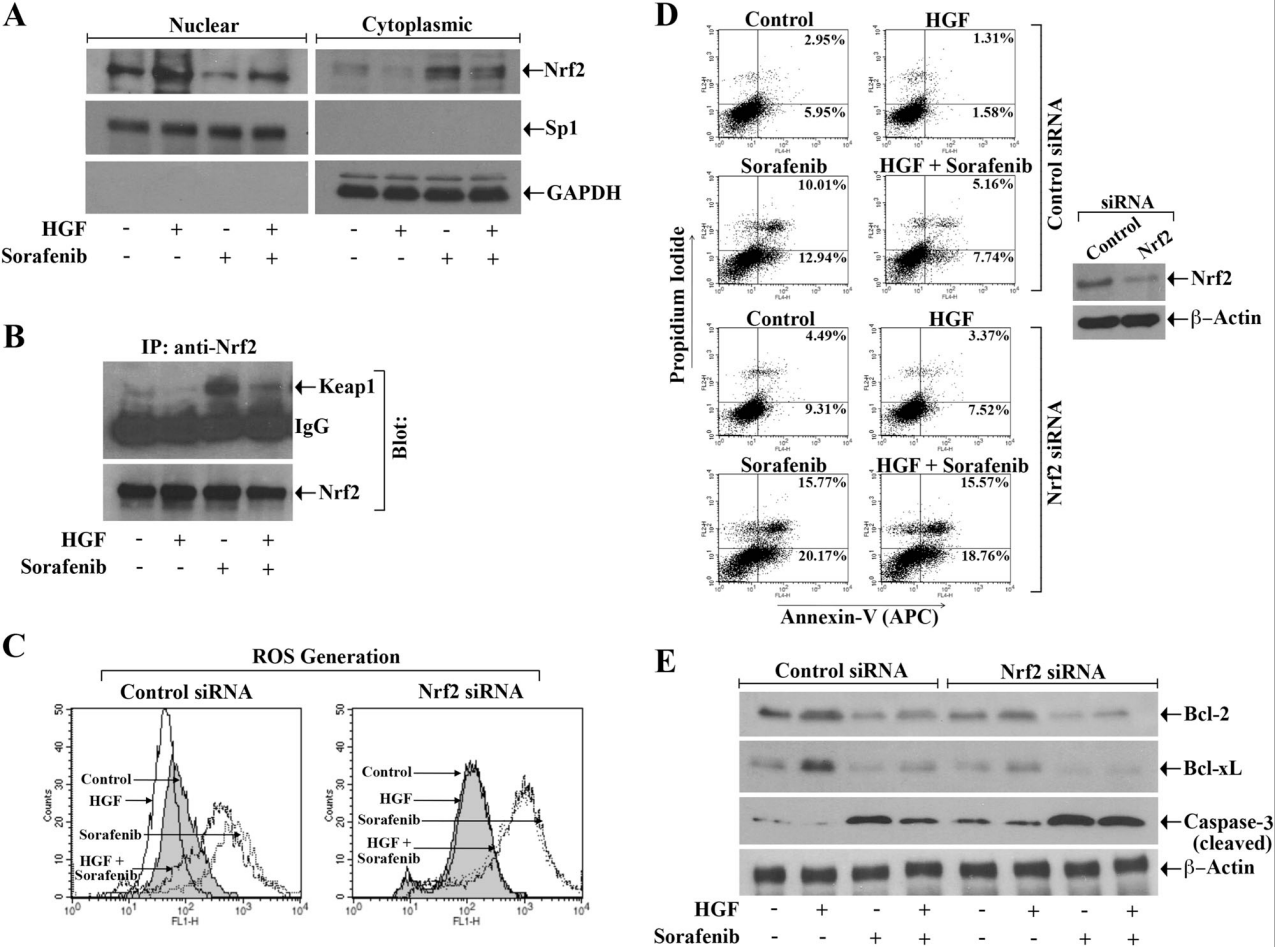
**c-Met activation induces the Nrf2 pathway; and silencing of Nrf2 attenuates the cytoprotective function of c-Met against sorafenib-mediated ROS generation and apoptosis of renal cancer cells.** **a**, **b** 786-O cells were incubated using combinations of HGF (50 ng/ml), sorafenib (10 μM), or only vehicle for 12–24 h. **a** Following isolation of nuclear and cytoplasmic fractions, Western blot was performed to check the level of Nrf2. Purities of nuclear and cytoplasmic fractions were examined through the expression of Sp1 and GAPDH respectively. **b** Cell lysates were immunoprecipitated (*IP*) with anti-Nrf2 antibody. The expression of Keap1 and Nrf2 in the immunoprecipitates were analyzed by Western blot. **c**–**e** After transfection with either Nrf2 siRNA (50 nM) or control siRNA for 48–72 h, 786-O cells were subjected to treatment (as in **a**) with combinations of HGF, sorafenib or vehicle for 24 h. **c** Cells were stained by using oxidative stress detection reagent and examined for endogenous reactive oxygen species (ROS) through flow cytometry. **d** The apoptotic cell death was checked through annexin V (APC) and propidium iodide stainings. The lysate of the transfected cells was analyzed for the expression of Nrf2 and β-Actin by Western blot *(right panel)*. **e** The cellular lysates were utilized for checking the levels of Bcl-2, Bcl-xL, cleaved Caspase-3, and β-Actin through Western blot. **a**–**e** Results are representative of three different experiments

Nrf2, which is over-expressed in renal cancer^29,30^, is one of the key transcription factors to regulate anti-oxidant HO-1^13,31^. Here, we evaluated how sorafenib in the absence or presence of HGF can modulate the localization of Nrf2 either in nucleus or cytoplasm. We observed that sorafenib treatment decreased the expression of nuclear Nrf2 and increased cytoplasmic Nrf2; however, in the presence of HGF, these events were markedly inhibited (Fig. 2a). As discussed earlier, in absence of oxidative stresses, Nrf2 activity is repressed by Keap1; while in the presence of oxidative stress, the Nrf2-Keap1 interaction is impaired and Nrf2 migrates to the nucleus^32,33^. We checked the status of Nrf2-Keap1 complex formation following sorafenib treatment and c-Met activation. Through immunoprecipitation studies, we found that although there was an increase in Nrf2-Keap1 complex in sorafenib-treated cells, it was markedly decreased following HGF treatment (Fig. 2b).

Next, we wanted to delineate the role of Nrf2 in c-Met-mediated protection of cancer cells against sorafenib-induced ROS generation and apoptosis. We observed that following knock-down of Nrf2, the HGF treatment could not reduce sorafenib-induced ROS generation as compared with control cells (Fig. 2c). Also, in Nrf2 knock-down cells, there was a marked increase in sorafenib-induced apoptosis (early + late); and the HGF treatment could not downregulate sorafenib-induced apoptosis (in Nrf2 knock-down cells) to the same level compared with control (Fig. 2d). We also checked the expression of pro- and anti-apoptotic markers in these cells. We found that sorafenib-induced attenuation of anti-apoptotic Bcl2 and Bcl-xL and induction of apoptotic cleaved caspase-3 were inhibited in the presence of HGF in control cells; however, following knock-down of Nrf2, the HGF treatment could not either increase Bcl2/Bcl-xL or decrease caspase-3 in sorafenib-treated cancer cells as compared with controls (Fig. 2e).

**Fig. 3:**
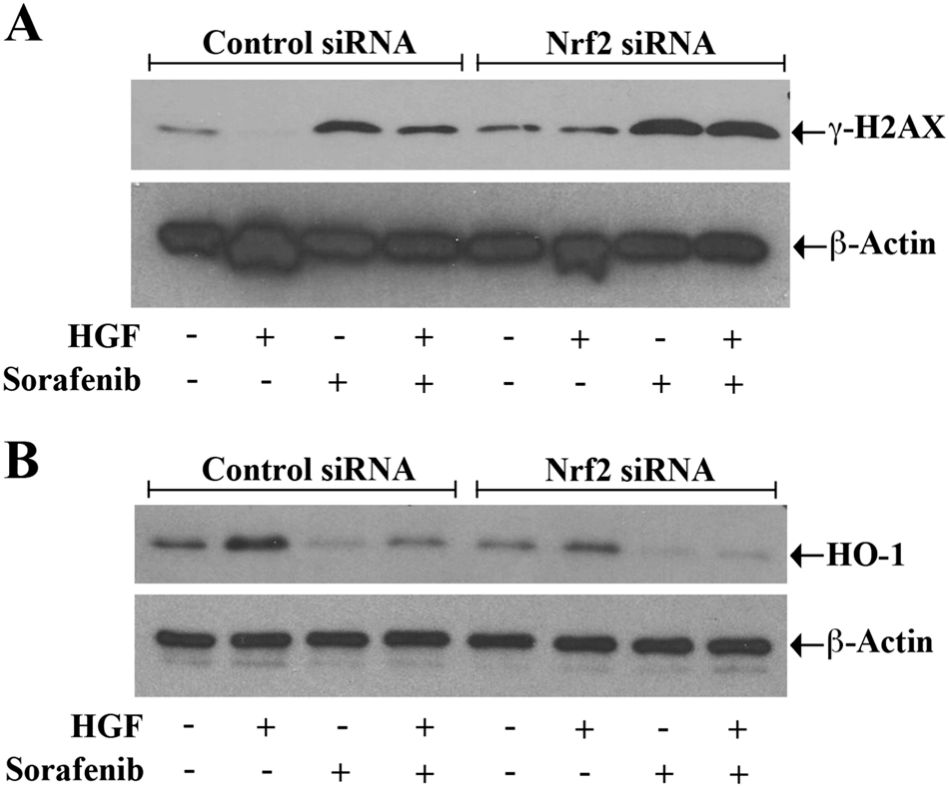
**Silencing of Nrf2 prevents the protective role of c-Met against sorafenib-induced DNA damage and the downregulation of anti-oxidant HO-1.** **a**, **b** 786-O cells were transfected with Nrf2 siRNA (50 nM)/control siRNA for 48–72 h. The transfected cells were subjected to combination treatments with HGF (50 ng/ml), sorafenib (10 μM), or only vehicle for 24 h. The cell lysates were utilized for checking the levels of the DNA damage marker γ-H2AX (**a**) and the redox protective enzyme HO-1 (**b**) β-Actin (internal control) by Western blot. β-Actin was measured as internal control. **a**, **b** Results are representative of three different experiments

Next, we studied how the DNA damage status of sorafenib-induced renal cancer cells is modulated following Nrf2 silencing by analyzing the expression of γ–H2AX. We observed that sorafenib treatment increased the expression of γ–H2AX, and it was decreased in the presence of HGF; however, when Nrf2 was knocked down, the HGF treatment failed to attenuate sorafenib-induced γ–H2AX overexpression as compared with control cells (Fig. 3a). Finally, we also examined the expression of anti-oxidant HO-1 in control and Nrf2 knocked down cancer cells after the sorafenib and HGF treatment. We found that in control cells, sorafenib-mediated downregulation of HO-1 was markedly prevented by HGF. In contrast, in Nrf2 silenced cells, sorafenib-induced decrease in HO-1 expression was not altered even after the HGF treatment (Fig. 3b). Altogether, these findings suggest that Nrf2 is one of the critical regulators for HGF-induced anti-oxidative cytoprotective functions in chemotherapeutic agent-treated cancer cells.

### Activation of c-Met inhibits mitochondrial as well as total ROS generation in RCC cells for their protection from sorafenib-induced apoptosis

**Fig. 4:**
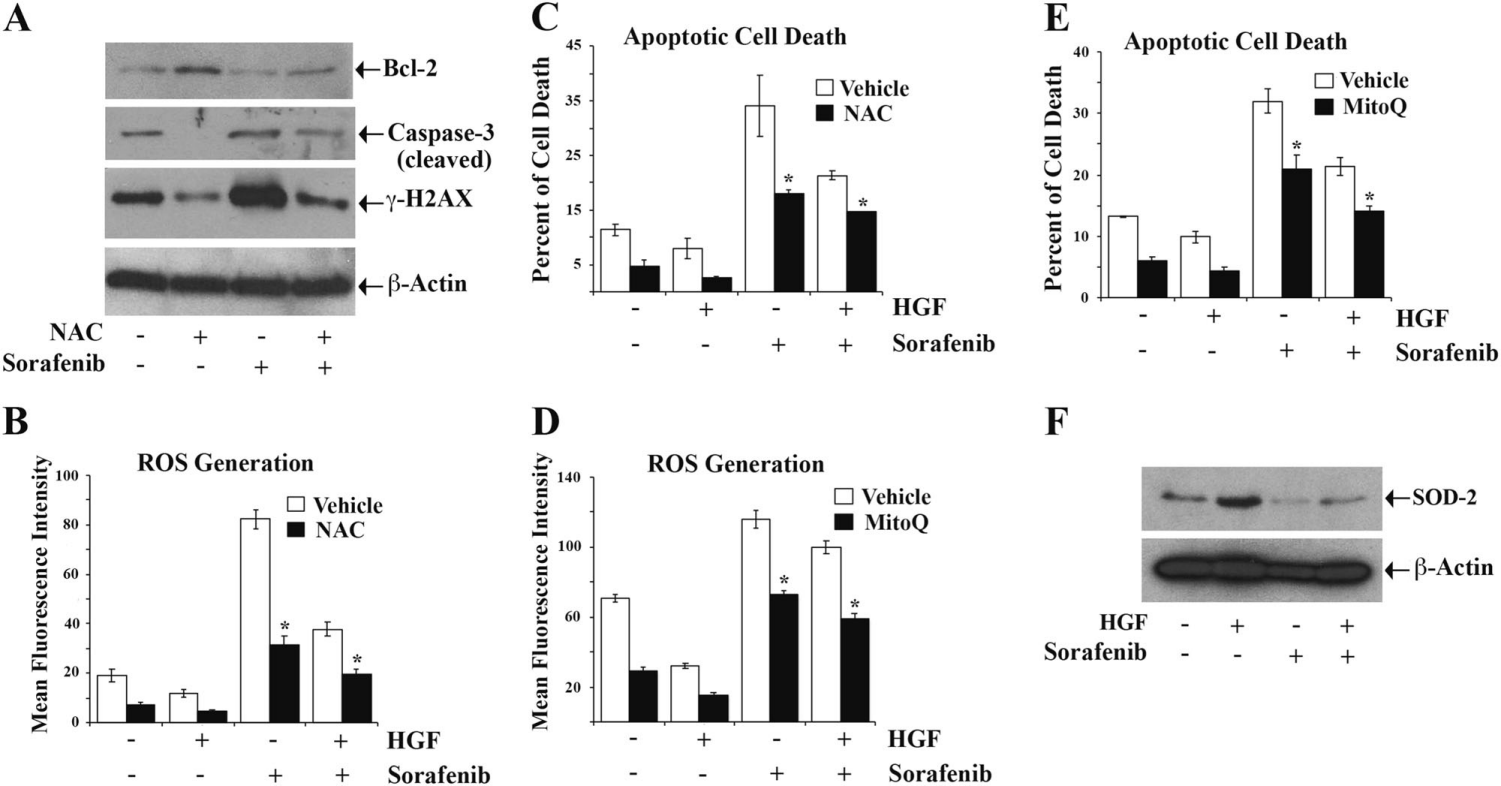
**Inhibition of reactive oxygen species (ROS) potentiates c-Met-mediated protection of renal cancer cells against sorafenib-induced apoptotic death.** **a** 786-O cells were pre-incubated with the ROS scavenger NAC (10 μM) for 2 h, and then subjected to treatment with sorafenib (10 μM) or vehicle for 24 h. The expression of Bcl-2, cleaved Caspase-3, γ-H2AX in cellular lysates was measured by Western blot. β-Actin was used as the internal control. **b** 786-O cells were pre-incubated with NAC (10 μM) for 2 h followed by combination treatments with HGF (50 ng/ml), sorafenib (10 μM) or only vehicle for 24 h. The cells were then stained by using oxidative stress detection reagent and checked for endogenous ROS through flow cytometry. The mean fluorescence intensity has been represented as bar graphs. **c** 786-O cells were subjected to treatment as in **b**, and then the percent of cell death was studied by using annexin V (APC) and propidium iodide staining through flow cytometry. The percent of total apoptotic cells (early + late) are represented as bar graphs. **d** Following incubation with the mitochondrial electron transport chain blocker Mitoquinone (MitoQ) (1 μM) for 2 h, 786-O cells were subjected to combination treatments with HGF (50 ng/ml), sorafenib (10 μM), or only vehicle for 24 h. The cellular ROS was then measured as described in **b**. **e** 786-O cells were subjected to treatment as in **d**, and then the percent of cell death was checked through annexin V (APC) and propidium iodide staining as shown in **c**. **f** 786-0 cells were subjected to combination treatments with HGF (50 ng/ml), sorafenib (10 μM), or only vehicle for 24 h, and then the expression of SOD-2 in cell lysates was measured by Western blot. β-Actin was checked as an internal control. **a**–**f** Results are representative of three different experiments. **b**–**e** The columns are the mean ± S.D. of duplicate readings from two independent experiments. *p *<* 0.05 compared with control cells treated with vehicle alone

As shown earlier, the activation of c-Met decreased ROS levels in renal cancer cells. Here, we utilized the effect of a cytoplasmic ROS scavenger NAC^26,27^ to evaluate the critical role of ROS in c-Met-mediated cytoprotection of cancer cells against sorafenib-induced apoptosis. First, we quantified the expression of pro- and anti-apoptotic markers and DNA damage marker in the presence NAC and Sorafenib. We observed that NAC pretreatment reduced the level of apoptotic cleaved caspase-3 and increased anti-apoptotic Bcl-2 in sorafenib-treated cells (Fig. 4a). Interestingly, NAC pretreatment significantly reduced the expression of the DNA damage marker γ–H2AX in sorafenib-treated cells (Fig. 4a). Next, the cells were treated with NAC, prior to HGF or sorafenib treatment. As expected, in the presence of NAC, both basal and sorafenib-induced ROS levels were decreased compared with control cells; and HGF treatment further decreased the levels of ROS in NAC pretreated cells (Fig. 4b). We also observed that sorafenib-induced apoptosis was significantly lower in cells pretreated with NAC, and it enhanced c-Met-mediated cytoprotection of RCC cells (Fig. 4c). These findings again suggest a major role of ROS in sorafenib-induced apoptotic cell death, and that the lower ROS levels can enhance c-Met-mediated cytoprotection.

In addition to cytoplasm, the electron transport chain of mitochondria represents the other important source of ROS^34^. Therefore, we analyzed the importance of mitochondrial ROS in sorafenib-induced cellular apoptosis and c-Met-mediated cytoprotection. The RCC cells were treated with the electron transport chain blocker Mitoquinone (MitoQ)^35^ prior to sorafenib or HGF treatment. We observed that MitoQ treatment reduced sorafenib-induced ROS generation compared with control cells; also, it also enhanced c-Met/HGF-mediated downregulation of ROS in sorafenib-treated cells (Fig. 4d). Furthermore, as shown in Fig. 4e, MitoQ treatment rendered the RCC cells less sensitive to sorafenib-induced apoptosis; and importantly, it enhanced c-Met-mediated cytoprotection against cellular apoptosis. Finally, we checked the expression of superoxide dismutase-2 (SOD-2), the intra-mitochondrial ROS scavenger^36^. We observed that sorafenib treatment decreased SOD-2 expression in renal cancer cells compared with control, while c-Met activation markedly restored SOD-2 in sorafenib-treated cells (Fig. 4f). Altogether, these results indicate that both cytoplasmic and mitochondrial ROS play critical role(s) in sorafenib-induced apoptosis of RCC cells; and the cytoprotection of cancer cells through c-Met activation involves the regulation of both forms of ROS.

### Downregulation of c-Met and HO-1 decreases renal tumor growth in vivo and promotes increased oxidative stress

**Fig. 5:**
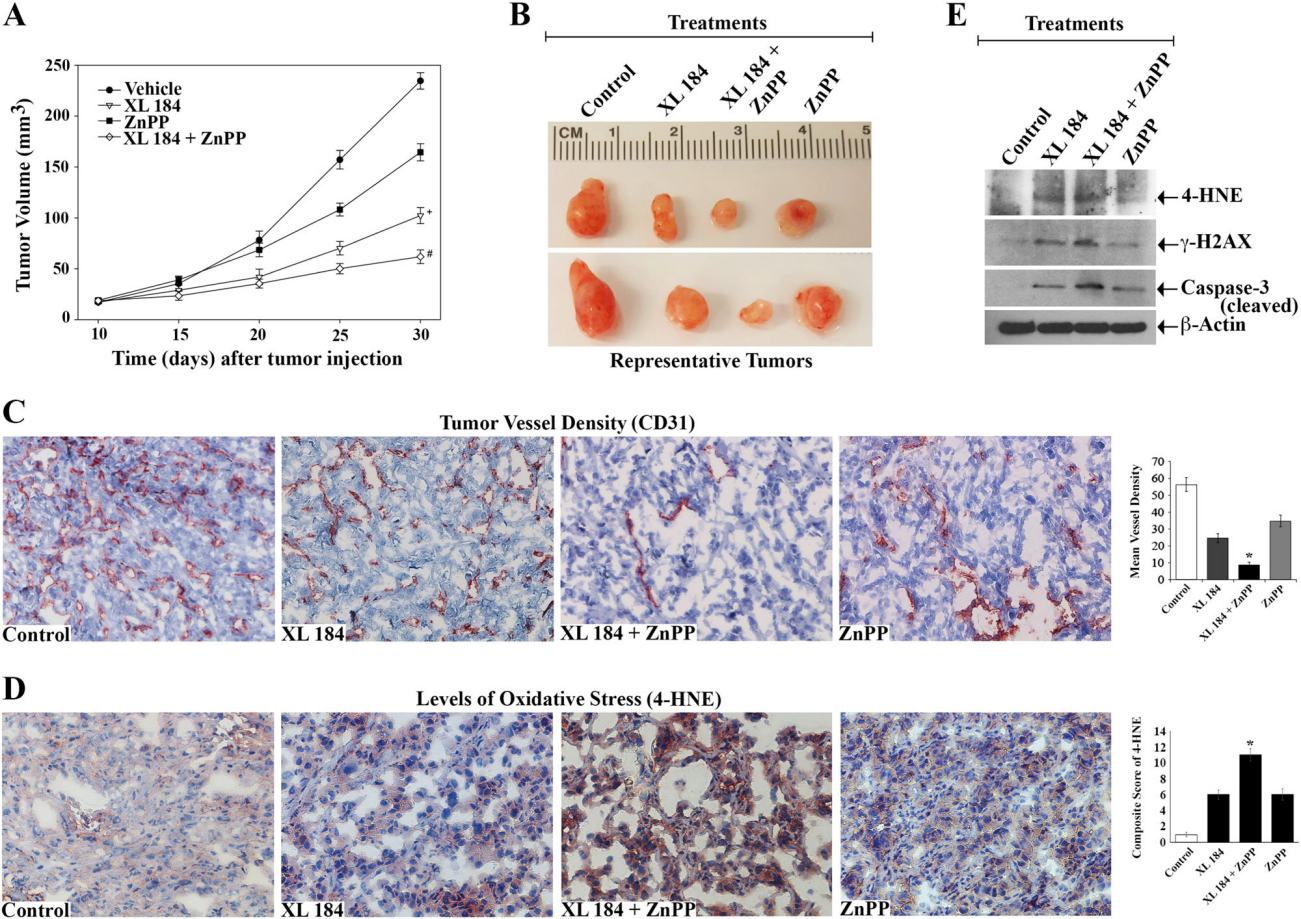
**Combination treatment with the inhibitors of c-Met and HO-1 decreases renal tumor growth through increased oxidative stress.** 786-O cells were injected subcutaneously in nude mice. When palpable tumors appeared (approximately in 10 days), animals (*n* = 5 each group) were injected intra-peritoneally with combinations of XL-184 (15 mg/kg/day) and ZnPP (25 mg/kg/twice a week); animals in control group were treated with vehicle. Treatments were continued for 20 days (i.e., 30 days following tumor injection). **a** Changes in the mean of tumor volumes have been shown in the tumor growth curve. The data reflect representative of two independent experiments. *Points*, average of tumor volume; *bars* ± S.D. + *p* *<* 0.001 compared with vehicle-treated group, and #*p* *<* 0.001 compared with ZnPP- and XL-184-treated groups. **b** Tumors were harvested on completion of the experiment, and two representative tumors from each experimental group has been presented to reflect tumor sizes. **c** Representative photomicrographs (magnification, x400) demonstrating the immunohistochemical expression of CD31 in the tissue sections from each treatment group. *Right*, mean vessel density analyzed by standard grid counting of CD-31-positive vessels at x400 magnification. The data reflect three different experiments, in which three to four non-overlapping fields of each specimen were examined in a blinded manner. *Columns*, average of vessel counts; *bars* ± S.D. *p *<* 0.05 compared with vehicle-/XL-184-/ZnPP-treated group. **d** Representative photomicrographs (magnification, x400) showing the immunohistochemical expression (brown-red stain) of the oxidative stress marker 4-Hydroxynonenal (4-HNE) in the harvested tissues from each treatment groups. *Right*, composite score of 4-HNE calculated as described in the methods section. The data reflect three different experiments, in which three to four non-overlapping fields of each specimen were examined in blinded manner. *Columns*, average of composite scores; *bars* ± S.D. *p *<* 0.05 compared with vehicle-/XL-184-/ZnPP-treated group. **e** Tissue lysates from the excised tumors of each treatment groups were used to measure the levels of 4-HNE, γ-H2AX, cleaved caspase-3 and β-actin through Western blot. Observations are representative of three different tissues from each group

Our earlier observations show that the activation of c-Met downregulates oxidative stress and apoptosis death of cancer cells through the modulation of Nrf2/HO-1 pathway^5,13^. To examine the physiological significance of our in vitro findings, we checked the effect of a c-Met inhibitor cabozantinib/XL 184 and a HO-1 inhibitor ZnPP on the growth and progression of renal tumor in vivo utilizing a xenograft model. As demonstrated, there was a marked decrease in tumor volume for the group of mice that was treated using a combination of XL-184 + ZnPP, compared with the vehicle-treated control group (Fig. 5a). The representative images reflecting tumor size from experimental groups after completion of the study is also presented (Fig. 5b). As the renal tumors are highly vascular, we checked the tumor vessel densities by using CD31 staining (Fig. 5c). We found that XL-184 + ZnPP combination treatment significantly decreased the tumor vessel density as compared with either the control group or the groups-treated with XL-184/ZnPP alone.

**Fig. 6:**
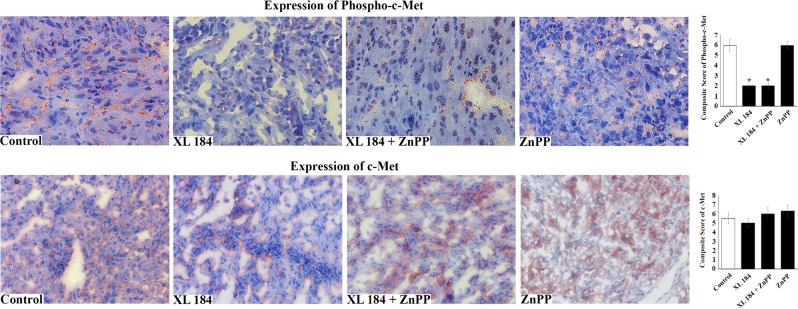
**Levels of phospho-c-Met and total c-Met in the tumor tissues from mice treated with different combinations of XL-184 and ZnPP.** Representative photomicrographs (magnification, x400) showing the immunohistochemical expression (red stain) of the phospho-c-Met (*upper panel*) and total c-Met (*lower panel*) in the tumor tissues from each treatment groups as described in Fig. 5. *Right*, composite score for phospho-c-Met/c-Met analyzed as described in the methods section. The data reflect three different experiments, in which three to four non-overlapping fields of each specimen were examined in blinded manner. *Columns*, average of composite scores; *bars* ± S.D. *p *<* 0.05 compared with vehicle-treated group

Next, we examined the level of the oxidative stress marker 4-hydroxynonenal (4-HNE) in the tumor tissues of different treatment groups. Interestingly, there was a marked increase in 4-HNE expression in the tumors of mice treated with a combination of XL-184 + ZnPP, compared with either the vehicle-treated group or the groups-treated with XL-184/ZnPP alone (Fig. 5d). Similar results were obtained for the expression of 4-HNE in the tumor tissue lysates as observed by Western blot (Fig. 5e, *top panel*). In addition, there was a marked increase in expression of the DNA damage marker γ–H2AX in tumors of the XL-184 + ZnPP-treated group (Fig. 5e, *top second panel*). Finally, we found that cleaved caspase-3 was markedly increased in tumor tissue lysates of the XL-184 + ZnPP-treated group compared with controls. We also confirmed through immunohistochemistry that phosho-c-Met was markedly downregulated in tumor tissues following XL-184 treatment; while, the expression of total c-Met did not change at significant level (Fig. 6).

Altogether, our findings in this tumor xenograft model suggest that the combined inhibition of c-Met and the HO-1 can significantly induce oxidative stress and apoptosis to restrict renal tumor growth.

## Discussion

c-Met, a receptor tyrosine kinase, which is over-expressed in renal cancer, plays a crucial role in tumor growth, survival, and progression. However, the mechanism(s) for c-Met-induced protective action against oxidative stress is not well studied. Here, we demonstrate that c-Met can induce the protection of renal cancer cells against oxidative stress through the modulation of redox protective Nrf2/HO-1 and downregulation of ROS.

Oxidative stress leads to an an elevated level of ROS; and harmonizing the cellular ROS is required for maintainiong normal physiological functions^34^. Increased ROS can lead to tumorigenesis ^37,38^; however, many cancer therapeutic agents, like sorafenib, can utilize the cellular ROS for their cytotoxic activity^23^. ROS can directly induce DNA damage, leading to cancer cell apoptosis^26–28,39^. A prime source of cellular ROS is the mitochondrial electron transport chain^34,40^. Enzymes, like NADPH oxidase (NOX)^11^, xanthine oxidase (XO)^41^, and lipoxygenase (LOX)^42^ are other principal sources of ROS. It has been shown that the induction of c-Met can reduce NOX4, p67phox, and p22phox expression in hepatocytes^43^. However, it is not clear how c-Met may regulate ROS generation and mediate chemoresistance in cancer cells.

In a recent report, Gillissen et al.^24^ demonstrated that sorafenib can induce a rapid change in the mitochondrial membrane potential accompanied by ROS generation, which leads to apoptotic death of cancer cells. In a clinical study, Coriat et al.^23^ showed that higher concentrations of advanced oxidation protein products (AOPP) in serum arising from oxidative stress are linked with an improved survival of cancer patient following sorafenib treatment. Cellular ROS scavenging can considerably reduce the cytotoxic activity of sorafenib. We observed that sorafenib-induced DNA damage and apoptotic death of renal cancer cells was indeed downregulated in presence of either the ROS scavenger NAC or the mitochondrial ROS inhibitor MitoQ, and also following c-Met activation. Superoxide dismutase-2 (SOD-2) is an intra-mitochondrial ROS scavenger; and it has been reported that there is higher SOD-2 expression in metastatic RCC patients^44^. Interestingly, we also observed that sorafenib can downregulate SOD-2 in cancer cells, and it is markedly prevented following c-Met activation. Thus, c-Met plays major part to relieve renal cancer cells from chemotherapeutic drug-induced oxidative stress. Similar to our findings, it has been shown that c-Met activation can also be associated with sorafenib resistance in hepatocellular carcinoma^45,46^.

In our previous studies^5,13,15^, we reported that the anti-oxidant cytoprotective molecule HO-1 is over-expressed in renal cancer; and c-Met can induce HO-1 through the transcription factor Nrf2, which is considered to be its master regulator. HO-1 promotes tumor growth through increased angiogenesis^47^ and decreased apoptosis^5,48^. We observed that the sorafenib treatment could promote ROS-induced oxidative stress in renal cancer cells through the inhibition of Nrf2-HO-1 cytoprotective pathway. However, in the present report, for first time we show that the induction of c-Met can markedly overcome sorafenib-mediated oxidative stress and DNA damage through the activation of Nrf2-HO-1. Nrf2 acts as a key player that neutralizes ROS and restores cellular redox balance. Cells can utilize different mechanism(s) to recognize oxidative stress through interference with the Keap1-Nrf2 complex formation. In clear cell as well as in papillary renal cell carcinoma, it has been reported that Nrf2 is highly active due to the loss of Keap1 functions that lead to increased accumulation of Nrf2 in the nucleus^29,30,49^. Here also, we observed that the induction of c-Met markedly decreases the sorafenib-induced complex formation between Keap1 and Nrf2 in renal cancer cells. Interestingly, the silencing of Nrf2 significantly attenuated the protective action of c-Met against sorafenib-induced oxidative stress, and increased cancer cell apoptosis. We showed the roles of Bcl-2, Bcl-xL and cleaved caspase-3 in regulating the anti-apoptotic function of c-Met in sorafenib-treated cells. However, some additional apoptotic/anti-apoptotic factors (Bax, Fas ligand and the inhibitor of apoptosis family members, IAPs) may also be involved in HGF-mediated survival of sorafenib-treated renal cancer cells^50–52^.

Although the effects of c-Met inhibition have been reported in different cancer types, no other studies have explored the potential therapeutic effect(s) of a combination treatment with a c-Met inhibitor and an inhibitor of its downstream effector HO-1 on renal cancer growth. In a pre-clinical model, we observed that a combination treatment with a c-Met inhibitor and a HO-1 inhibitor ZnPP markedly decreased the growth of renal tumors with reduced blood vessel densities; and it is associated with markedly increased oxidative stress, DNA damage and apoptotic markers within the tumor tissues. For the first time, we show that the combination treatment increased the expression of 4-HNE, which is a critical byproduct of intracellular oxidative stress. It is a lipid electrophile that can form covalent complexes with proteins, nucleic acids, and membrane lipids to initiate several signaling cascades leading to cell death^7,53^. Thus, the therapeutic strategies that can increase the oxidative stress of renal tumors can have potential clinical significance. Also, in future studies, it will be interesting to check the in vivo relevance of c-Met-HO-1 inhibition on sorafenib-induced oxidative stresses.

In summary, our observations suggest that the c-Met-Nrf2-HO-1 pathway plays a critical part in regulating the oxidative stress of renal tumors. Novel combination therapies that target c-Met and Nrf2/HO-1 together, can promote ROS-induced oxidative stress; and this can facilitate the cytotoxic potential of chemotherapeutic agents in mediating apoptotic death of cancer cells. Altogether, our findings clearly have pathophysiological significance with translational importance.

## Materials and methods

### Reagents

Sorafenib and Cabozantinib/XL-184 were purchased from Selleckchem. Zinc Protoporphyrin (ZnPP) was obtained from Frontier Scientific. The recombinant human HGF was purchased from Peprotech. Gene-specific siRNAs for Nrf-2(NFE2E2) along with control were obtained from Qiagen. The cells were subjected to transfection with the siRNA by using Lipofectamine 2000 (Invitrogen). The ROS scavenger N-acetylcysteine (NAC) was purchased from Sigma-Aldrich. The Mitochondrial ROS blocker Mitoquinone (MitoQ) was obtained from Focus Biomedicals.

### Cancer cell lines

786-O and ACHN, two renal cancer cell lines isolated from human tumors, were purchased from ATCC. The cells were grown using RPMI 1640 medium that was supplemented with 10% fetal bovine serum (Invitrogen). For treatment with HGF, the cells were serum starved for 12 h. Both the cell lines have been authenticated through short tandem repeat (STR) profiling from ATCC within last 2 years, and all of them were mycoplasma free.

### Western blot analysis

A total of 2 × 10^6^ cells were seeded in 100 mm dish. Cells were treated with RIPA buffer (Cell Signaling) for lysis. Proteins (100 μg) were separated through SDS-polyacrylamide gel, followed by transfer to polyvinylidene difluoride (PVDF) membrane (Millipore Corp). PVDF membranes were coated with anti-HO-1 (Cat# 5061), anti-Nrf2 (Cat# 12721), anti-Keap1 (Cat# 4678), anti-Bcl-xL (Cat# 2764), anti-Bcl-2 (Cat# 2876), anti-GAPDH (Cat# 5174), anti-cleaved Caspase-3 (Cat# 9661), anti-γH_2_AX (Cat# 2577), anti-SOD-2 (Cat# 13141), anti-Sp-1 (Cat# 5931), or anti-β-actin (Cat# 3700) (all from Cell Signaling); and then incubated in the presence of peroxidase-linked (HRP) secondary antibody. Protein bands were visualized through chemiluminescent substrate (Pierce)^5,15^.

### Isolation of nuclear fractions

A total of 0.8 × 10^6^ cells were seeded in 60 mm dish. Nuclear extracts from renal cancer cells were isolated by utilizing a kit for nuclear extraction (Active Motif) following protocol of the manufacturer.

### Immunoprecipitation assays

A total of 2 × 10^6^ cells were seeded in 100 mm dish. Immunoprecipitation was performed using 0.5 mg of cellular proteins in excess antibody with anti-Nrf2 (Cat# 12721, Cell Signaling). The immunocomplex was captured using protein A-Sepharose beads (GE Healthcare), and precipitated proteins were analyzed by western blot using anti-Keap1 (Cat# 4678, Cell Signaling).

### Cell proliferation assay

A total of 0.1 × 10^5^ cells were seeded in every well of a 96-well plate. Cell proliferation was analyzed utilizing the MTT cell proliferation assay kit (ATCC)^15^.

### Apoptosis assay

A total of 0.5 × 10^5^ cells were seeded in each well of a six well culture plate. Apoptotic cell death was assessed through Annexin-V and propidium Iodide (PI) staining using an allophycocyanin (APC)-conjugated kit to detect apoptosis (eBioscience). Stained cells were studied through flow cytometry on a FACSCalibur^5,15^.

### Detection of reactive oxygen species (ROS)

A total of 0.5 × 10^5^ cells were seeded in each well of a six-well culture plate. Endogenous ROS were analyzed utilyzing a kit to detect total ROS (Enzo Life Sciences) following protocol of the manufacturer. This kit has been designed to check real-time ROS generation in live cells through flow cytometry.

### Formation of tumor in a xenograft model in mice

Animal studies were undertaken following guidelines according to the Institutional Animal Care and Use Committee (IACUC) protocol that has been approved through Boston Children’s Hospital. A total of 1 × 10^6^ 786-O cells per mice were subcutaneously injected in flanks of 6-weeks-old male immunodeficient (nude) mice (NU/J, Jackson Laboratory, Bar Harbor, ME). After the palpable tumors were noticed, animals (*n* = 5 each group) were randomly distributed (in a blind-folded manner) to different groups for treatment. Before injection, XL-184 and ZnPP were diluted in normal saline to get the required concentration. Mice were treated intra-peritoneally with combinations of XL-184 (15 mg/kg/day) and ZnPP (25 mg/kg/twice a week); while the mice in control group were injected only with vehicle. Treatments were continued till day 20 (30 days following injection of tumor). Size of the tumors was measured by digital caliper. The volume was calculated through standard method, with the formula *V* = *π*/6 x *a*^2^ x *b* where *a* is the short and *b* is the long axis of the tumor^15^. The tumors were harvested and subjected to routine processing for molecular analyses.

### Immunohistochemistry

Harvested tumor tissues were quickly frozen using the OCT compound (Tissue-Tek, Torrance, CA). Tissue sections were prepared on a cryostat (Leica, Buffalo Grove, IL). For immunolabeling, the sections were then treated with anti-CD31 (1:50 dilution) (Cat# 550274, BD Pharmingen)), anti-phospho-c-Met (1:300 dilution) (Cat# 3077), anti-total Met (1:300 dilution) (Cat# 8198, Cell Signaling), or anti-4-HNE (1:200 dilution) (Cat# ab46545, Abcam); and subsequently labeled with species-specific horseradish peroxidase-conjugated secondary antibody. Tissue specimens were developed in 3-amino-ethylcarbazole and counterstained with Gills hematoxylin. In anti-CD31-stained sections, the means for vessel densities were calculated by the standard method of grid counting at a magnification of x400^5,15^.

The samples were quantitatively analyzed by the software ImageJ using an immunohistochemical composite scoring system or the immunoreactive scoring system (IRS)^54^. In this quantification system, the composite score or immunoreactive score provides a range of 0 to 12 as a product of percentage of positive cells score (0–4) and staining intensity score (0–3); and a final score of 9–12 is considered to be strongly positive expression.

### Statistics

Changes in mean volume of the tumors were compared through two-way repeated-measures analysis of variance (ANOVA) for checking the slope differences among experimental groups over the period of time using the group-by-time interaction *F*-test. Data are represented through mean with standard deviation (SD). The power analysis suggested that a sample size of *n* = 5 mice per experimental group can generate 80% statistical power (two-tailed *α* = 0.05, *β* = 0.20) to get significant differences of 50% or more in tumor volume among treatment groups. Conservative two-tailed values of *p* < 0.01 were taken as statistically significant for protecting against type I errors when comparing many groups. In case of in vitro studies, statistical significances were calculated through Student’s *t*-test. Any differences with *p* < 0.05 were taken as significant. We fixed sample sizes considering the variation and mean of the samples. No statistical method was utilized to predetermine sample sizes. No samples were excluded from any analysis.

## Supplementary information

Supplementary Figure-1

Supplementary Figure-2
