# Supplementary material for: Activation of c-Met in cancer cells mediates growth-promoting signals against oxidative stress through Nrf2-HO-1
Source: Oncogenesis. 2019 Jan 15;8(2):7. doi: 10.1038/s41389-018-0116-9 (PMC6333845; doi:10.1038/s41389-018-0116-9)
Supplement: Supplementary file 1 — Supplementary Figure-1 [file 41389_2018_116_MOESM1_ESM.pdf]

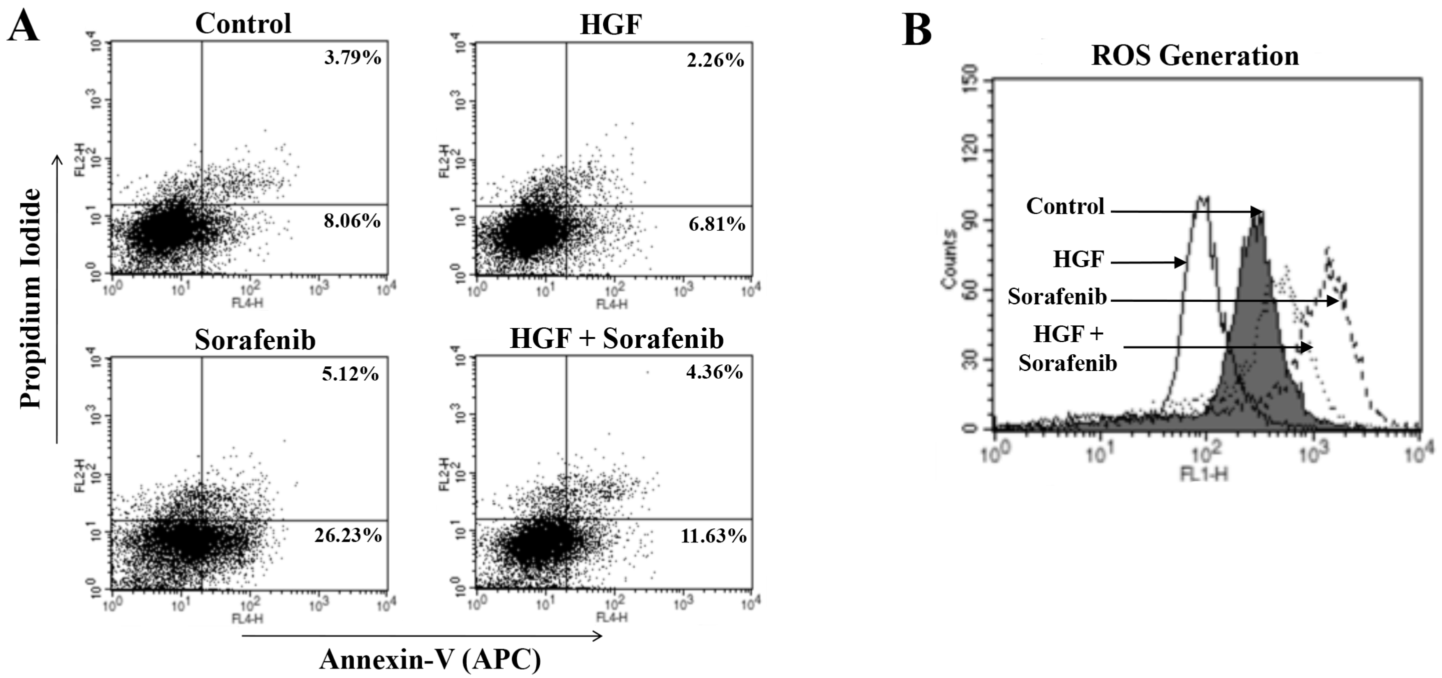

Supplementary Figure-1

HGF treatment decreases sorafenib-mediated ROS generation and apoptosis in ACHN renal cancer cells: Cells were treated with different combinations of HGF (50 ng/ml), sorafenib (10 uM) or vehicle alone for 24 hours. (A) Apoptotic index of the cells was determined by annexin V (APC) and propidium iodide staining through flow cytometry. (B) Following treatment, cells were stained with oxidative stress detection reagent and analyzed for cellular ROS by flow cytometry as described in Materials and Methods.
