# Supplementary material for: Activation of c-Met in cancer cells mediates growth-promoting signals against oxidative stress through Nrf2-HO-1
Source: Oncogenesis. 2019 Jan 15;8(2):7. doi: 10.1038/s41389-018-0116-9 (PMC6333845; doi:10.1038/s41389-018-0116-9)
Supplement: Supplementary file 2 — Supplementary Figure-2 [file 41389_2018_116_MOESM2_ESM.pdf]

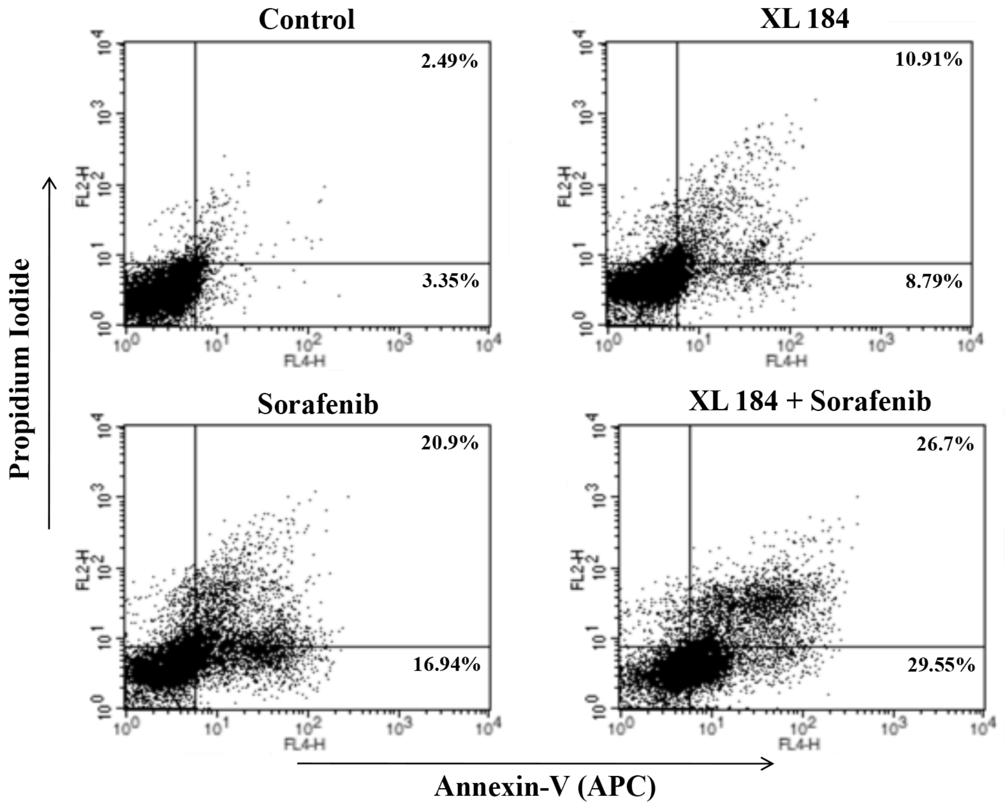

Supplementary Figure-2

Inhibition of c-Met by XL 184 treatment promotes sorafenib-mediated apoptosis of 786-O renal cancer cells: Cells were first pre-treated with XL 184 (10 uM) for 2 hours, and then treated with sorafenib (10 uM) for 24 hours. Apoptotic index of the cells was determined by annexin V (APC) and propidium iodide staining through flow cytometry.
